# Tinea Capitis by *Microsporum canis* in an Elderly Female with Extensive Dermatophyte Infection

**DOI:** 10.1007/s11046-020-00519-9

**Published:** 2021-01-26

**Authors:** Zhihui Yang, Wei Chen, Zhe Wan, Yinggai Song, Ruoyu Li

**Affiliations:** 1 Department of Dermatology and Venerology, Peking University First Hospital, No. 1 Xi’anmen Street, Xicheng District, Beijing, 100034 People’s Republic of China; 2 Research Center for Medical Mycology, Peking University, Beijing, People’s Republic of China; 3 Key Laboratory of Molecular Diagnosis of Dermatoses, Peking University First Hospital, Beijing, People’s Republic of China; 4 National Clinical Research Center for Skin and Immune Diseases, Beijing, People’s Republic of China

**Keywords:** Adult tinea capitis, Extensive dermatophyte infection, *Microsporum canis*, Terbinafine

## Abstract

Tinea capitis is a type of dermatophyte infection primarily affecting children. We report a case of an elderly woman with well-controlled diabetes mellitus presenting with a six-month history of erythema with yellow crusts on her scalp and extensive erythematous patches with scales on the body skin. She adopted a stray cat before the disease onset. Dermoscopic findings and manifestation under the Wood’s lamp favoured the diagnosis of tinea capitis. Further microscopic examinations of her scalp, including direct KOH and fluorescence stain examination, fungal culture and polymerase chain reaction sequencing identification confirmed the diagnosis of tinea capitis caused by *Microsporum canis*. Treatment with oral terbinafine was effective. Adult tinea capitis is often misdiagnosed due to its rarity and atypical presentation. However, in some regions, the incidence of tinea capitis in immunocompetent adults is rising which requires the awareness of clinicians. A thorough history (including the animal contacting history), physical examination and further mycological examinations are required for diagnosis. *Trichophyton violaceum* is the most common dermatophyte species in most regions while adult tinea capitis caused by *Microsporum canis* is less common. Terbinafine, griseofulvin and itroconazole have been reported to be effective drugs for the treatment of tinea capitis, and terbinafine can be considered as systemic treatment in elderly patients with comorbidities to reduce the drug–drug interaction.

## Introduction

Dermatophyte infections are commonly distributed worldwide. According to different clinical manifestations, they are classified into tinea capitis, tinea corporis, tinea cruris, tinea pedis, Majocchi’s granuloma and tinea unguium (dermatophyte onychomycosis). Among them, tinea capitis primarily occurs in children, and extensive tinea corporis occurs mainly in patients with underlying immune disorders such as HIV infection, systematic and topical use of steroids [1, 2]. Here, we report a case of tinea capitis caused by *Microsporum canis* (*M.canis*) with extensive superficial dermatophyte infection in an elderly female with well-controlled diabetes mellitus.

## Case Report

**Fig. 1:**
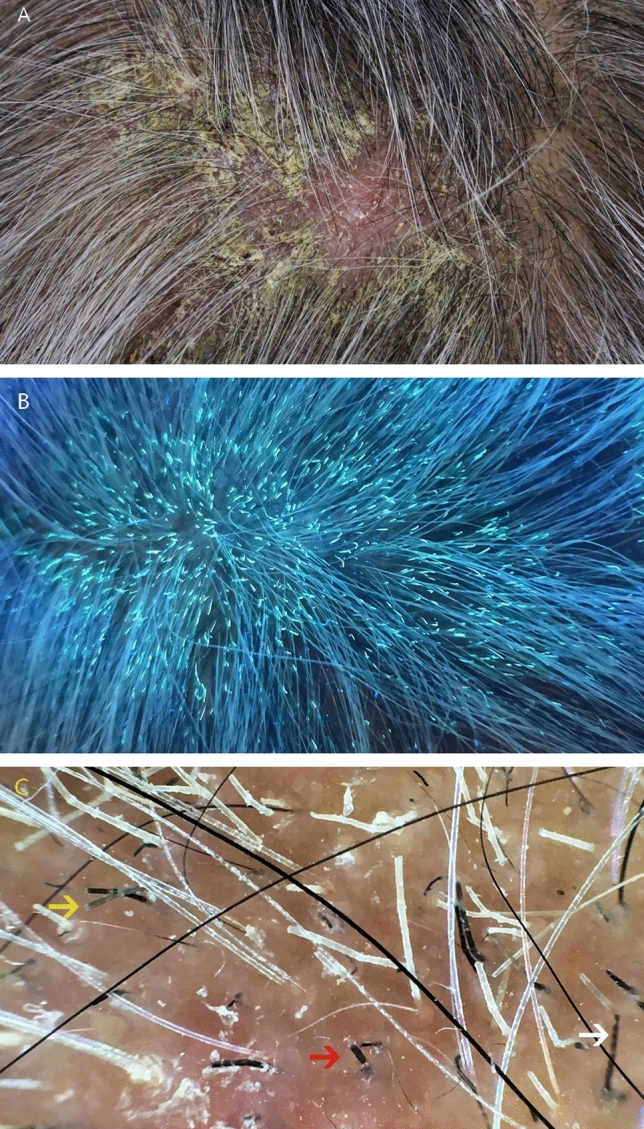
**a** Scalp lesions presenting as multiple scaly erythematous patches with thick grayish-yellow crusts. **b** Broken hair with bright-green fluorescence under the Wood’s light illumination. **c** Morse code-like hairs (white arrow), white sheath-surrounded hairs (yellow arrow) and short broken hairs (red arrow) under the dermoscope (× 20)

**Fig. 2:**
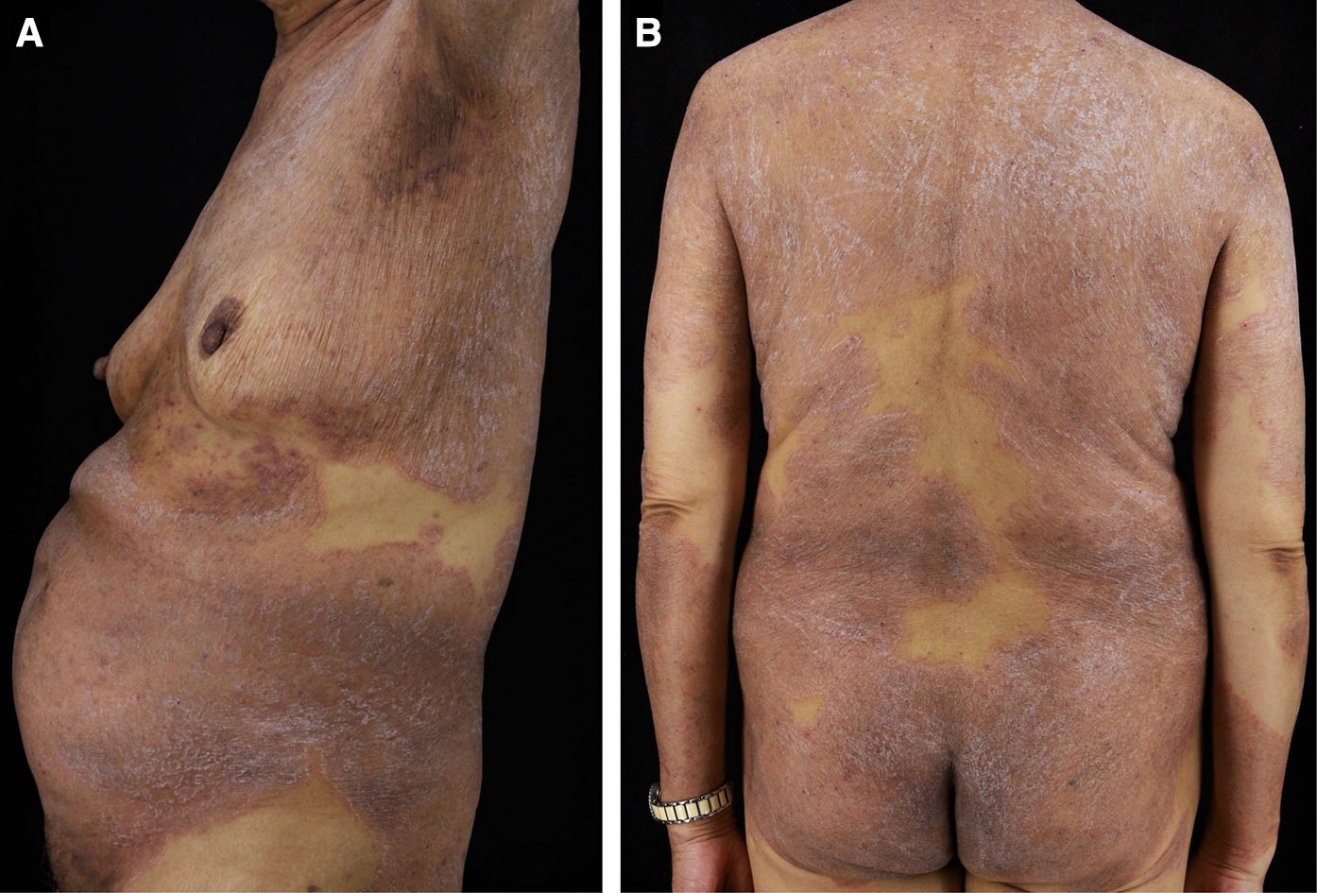
**a**, **b** Extensive erythematous patches on the trunk and arms, with well-demarcated borders and considerable white superficial scales

A 71-year-old woman presented with a six-month history of persistent scalp rashes together with generalized body skin lesions. The rashes first involved her chest skin and then gradually spread to the whole trunk, scalp, groin and all extremities with severe pruritus and malaise. She was diagnosed as seborrhoeic dermatitis and psoriasis previously and treated with compound econazole nitrate and triamcinolone acetonide cream without improvement. She had diabetes mellitus for nearly 5 years. Her fasting blood glucose was controlled within 6–7 mmol/L and postprandial blood glucose within 8–9 mmol/L with subcutaneous insulin. She had been diagnosed with onychomycosis for 2 years without treatment. Physical examination revealed multiple scaly erythematous patches on her scalp with thick greyish-yellow crusts and patches of alopecia with broken hair (Fig. 1a). Bright-green fluorescence was prominent on her hair under Wood’s light illumination (Fig. 1b). Morse code-like hairs, white sheaths and short broken hairs could all be seen under a dermoscope (Fig. 1c). Multiple large erythematous patches could be seen on her face, neck, bilateral ears, trunk, extremities, as well as skin folds including perineum, groin and axillae (Fig. 2a, b). These patches showed elevated and well-demarcated borders, with considerable white superficial scales. All of her toenails revealed discoloration and subungual hyperkeratosis. There was no associated lymphadenopathy.

**Fig. 3:**
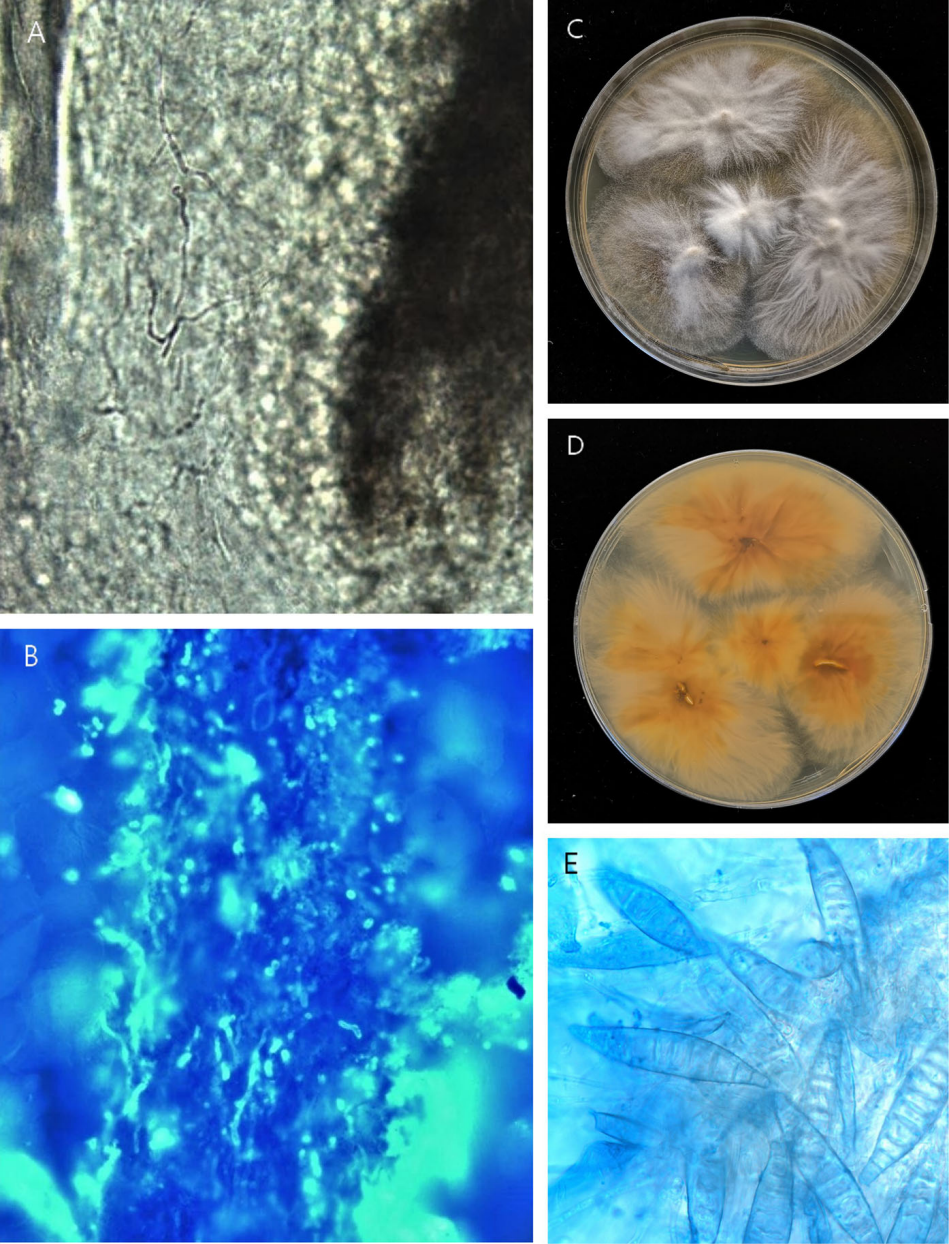
**a**, **b** Ectothrix hyphae and spores revealed in the direct KOH examination and fluorescence stain. **c, d** Gross morphology of *M.canis* on Sabouraud dextrose agar cultured from the hair of both the patient and the cat (after 10-day incubation at 28 °C). The cottony colony is white from the front and orange from the reverse. **e** Microscopic examination (× 40) showing septate hyphae and typical macroconidia of *M.canis*

Direct microscopic examinations (including 10% KOH smear and fluorescence stain) of broken hair revealed ectothrix hyphae and spores (Fig. 3a, b). The direct KOH examination of skin scraping on her trunk and subungual debris were also positive for fungal hyphae. Further history taking revealed contact to a stray cat before the onset of disease. Fungal cultures on Sabouraud dextrose agar (SDA) of both scraping from the patient scalp and the adopted stray cat hair grew *Microsporum canis* (Fig. 3c, d, e). Unfortunately, fungal cultures of toenails failed to grow any fungal colonies. The sequencing internal transcribed spacer (ITS) rDNA region with ITS1/ITS4 primer further confirmed the strain identification results and the homology of the strain from the patient and cat.

**Fig. 4:**
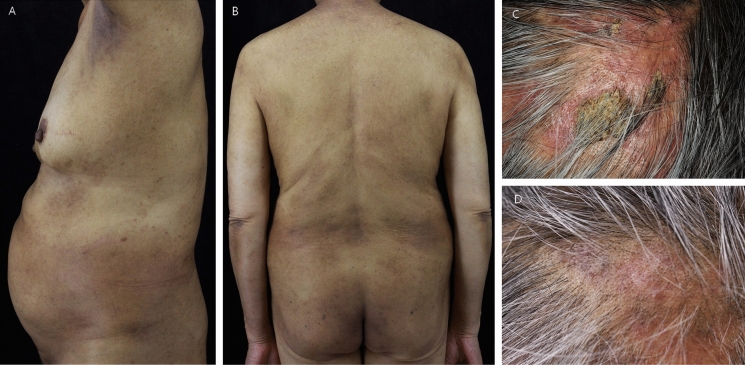
**a**, **b** Lesions on the trunk and arms cleared after two-week treatment. **c, d** Scalp lesions relieved after two-week treatment and cleared after six-week treatment

Considering the coexistence of tinea capitis, extensive tinea corporis, tinea pedis and onychomycosis, the patient was diagnosed as tinea capitis caused by *M.canis* with extensive dermatophyte infection. She was treated with oral terbinafine 250 mg daily, topical bifonazole solution and ketoconazole cream on her body twice per day. Two weeks later, the rashes on trunk and extremities cleared both clinically and mycologically (Fig. 4a, b). As for her scalp lesion, it took her 6 weeks to be cleared clinically (Fig. 4c, d) and to turn negative mycologically, but 8 weeks for the culture of scrapings and 12 weeks for the result of Wood’s lamp to turn negative, respectively. Her toenails finally got normal gradually in the following 4 months with the continuing treatment of oral terbinafine.

## Discussion

Tinea capitis is a type of fungal infection on the scalp, which primarily affects children aged 3–7 years old [3]. Tinea capitis is uncommon in adults, due to the pH changes and fatty-acids increase in the adult scalp [4]. The proportion of adults among tinea capitis patients was reported to be 2.9% and 4.2% in multi-centre studies from Mexico [5] and Egypt [6], respectively. In single-centre studies, this proportion varies significantly in different countries, from 1.5 to 44.3% [7–11]. In mainland China, adults took up 6.0–13.6% of the tinea capitis population in the 1980s–1990s [12]. This number remained to be 9.0% in the 21th century [13]. In contrast, up to 63% of tinea capitis patients were reported to be adults in a single centre of Taiwan, China [14]. These indicate that adult tinea capitis is becoming less uncommon in some regions, especially in postmenstrual elder women, due to their reduced secretion of fungistatic sebum after menopause. In adult patients, the female-to-male ratio was reported to be 2.2–5.4:1 [4–6, 11, 13, 15] and 26.7–93.5% of the female adults were postmenopausal [10, 11]. Apart from severe immunosuppressive diseases such as HIV infection and post-transplantation, systemic chronic disease including HCV infection (34.4%) and diabetes mellitus (22.4%) has been reported to associate with tinea capitis [6]. Systematic and topical use of corticosteroids are also risk factors [11, 16]. Close contact with animals was reported in 17.1–19.3% of the patients [6, 11, 13]. Furthermore, 20–72% [9, 10, 13] of the adult patients were reported to be immunocompetent. Due to the rise in the occurrence of tinea capitis in immunocompetent adults, adult tinea capitis needs the awareness of clinicians.

Causative agents of adult tinea capitis also vary across geological regions. *Trichophyton violaceum* was reported to be the main dermatophyte in adults in most countries including Egypt (56.9%, 33/58) [6], Iran (32.0%, 8/25) [10], southern Spain [9] and southern Taiwan, China (74%) [14]. Though *M. canis* is a major agent causing tinea capitis in children, it is less common in adults [17]. It was reported to account for 15.5% (9/58) infection in Egypt [6] and 16.0% (4/25) in Iran [10]. Exceptionally, *M. canis* caused 56.5% (13/23) of the adult cases in a single centre in Korea [11]. In mainland China since 2000, *Trichophyton violaceum* (35.2%, 70/199) was the most common agents, followed by *M. canis* (21.1%, 42/199), *Trichophyton mentagrophyte* (16.1%, 32/199) and *Trichophyton rubrum* (11.5%, 23/199) [13]. Other common dermatophytes isolated in adult patients included *Trichophyton tonsurans* [5], *Trichophyton verrucosum* and *M. gypseum* [6].

Fungal coinfection occurs in 60% of the tinea capitis patients [10]. However, tinea capitis caused by *M. canis* coexisting with extensive tinea corporis in adults is rare. In this case, the patient had well-controlled diabetes mellitus and denied any other immunosuppression conditions. We hypothesized that *M. canis* transmitted from animals had stronger pathogenicity which made the skin lesion so generalized. The delay in diagnosis and application of topical corticosteroids also contributed to the lesion generalization. However, the transmission ability of *M. canis* is weak without animal reservoirs, thus can hardly spread widely among the human population. Its virulence can be lost after about four human-to-human transmissions [3]. This characteristic can be reflected in this case, as the patient did not report any affected family members.

Adult tinea capitis mainly presents as seborrhoeic dermatitis with scaling, grey patch and kerion celsi [11]  or pseudo-alopecic plaques [5]. Due to its rarity and atypical presentation, 73.2% of the patients were misdiagnosed during their first consultation in one Korean hospital [11]. Seborrhoeic dermatitis is the diagnosis most commonly confused with tinea capitis in adults [11, 13]. Folliculitis is another major differential diagnosis [4, 11, 13]. The patient in this case was previously misdiagnosed as psoriasis and seborrhoeic dermatitis as these two diseases often present with similar erythema involving scalp and other body areas simultaneously. Thus, a detailed history taking including animal contact and a careful physical examination including searching for broken hair are required. Examinations including Wood’s lamp and dermoscopy can also help in diagnosis and monitoring the treatment effect [18]. Common dermoscopic findings may involve broken hairs, scales, black dots, perifollicular erythema, comma hairs, empty follicles and pustules [11]. In ectothrix infection caused by *M.canis*, Morse code-like hairs (AKA barcode-like hairs) [19, 20] and white sheaths [21, 22] are typical characteristics under the dermoscope. For a definitive diagnosis, mycological examinations are very important. Among them, 10% KOH microscopic examination of fungal elements is basic for diagnosis and treatment assessment of superficial fungal infection, and novel fluorescent staining can improve the detection rate [23]. Fungal culture can direct the antifungal therapy choice by identification of the dermatophyte.

Treatment of tinea capitis in adults is similar compared with children, yet the age and comorbidities of the patient need to be considered [18]. Terbinafine, griseofulvin and itraconazole have been widely used in the treatment of tinea capitis [10]. As terbinafine hardly influences cytochrome P450 and CYP3A4, it has fewer drug interactions and is increasingly used especially in older patients with multimorbidity [24]. In a meta-analysis comparing the efficacy of griseofulvin and terbinafine in tinea capitis, terbinafine was found to be more effective than griseofulvin for infection by *Trichophyton* sp., while griseofulvin was shown to be more effective than terbinafine for *Microsporum* sp. [25]. As *M. canis* can be resistant to the usual dose of terbinafine (250 mg daily), treatment longer than 4 weeks may be needed for a successful clinical and mycological response [26]. A topical antifungal agent is also advisable for 2-3 months [18]. In this case caused by *M.canis*, prolonged treatment with oral terbinafine was adopted considering the age and comorbidities of the patient, and the coexisting refractory onychomycosis.

Tinea capitis is not common in adults in most areas. Adult tinea capitis caused by *M.canis* and co-occurrence with extensive dermatophyte infection are both rarer. This case highlights the challenge in the diagnosis of tinea capitis with extensive dermatophyte infection in adults due to its rarity. In cases presenting as extensive erythema with scales on both scalp and body skin, the fungal infection should be considered. A thorough history including animal contact and physical examination searching for broken hairs are required. Dermoscope, Wood’s lamp examination and further mycological examinations are needed for diagnosis. Terbinafine can be considered as systemic treatment in elderly patients with comorbidities.

## Compliance with Ethical Standards

This case report was written complying with the checklist of essential elements instituted by *Mycopathologia* to guarantee the quality of published case reports [27].

## References

[1] Balighi K, Lajevardi V, Barzegar M, Sadri M (2009). Extensive tinea corporis with photosensivity. Indian J Dermatol.

[2] Metkar A, Joshi A, Vishalakshi V, Miskeen AK, Torsekar RG (2010). Extensive neonatal dermatophytoses. Pediatr Dermatol.

[3] Ginter-Hanselmayer G, Weger W, Ilkit M, Smolle J (2007). Epidemiology of tinea capitis in Europe: current state and changing patterns. Mycoses.

[4] Rebollo N, López-Barcenas AP, Arenas R (2008). Tinea capitis. Actas Dermosifiliogr.

[5] Medina D, del Carmen Padilla M, Fernández R, Arenas R, Bonifaz A (2003). Tiña de la cabeza en adultos: estudio clínico, micológico y epidemiológico de 30 casos en ciudad de México. Piel.

[6] El-Khalawany M, Shaaban D, Hassan H, AbdAlSalam F, Eassa B, Abdel Kader A (2013). A multicenter clinicomycological study evaluating the spectrum of adult tinea capitis in Egypt. Acta Dermatovenerol Alp Pannonica Adriat.

[7] Duarte B, Galhardas C, Cabete J (2019). Adult tinea capitis and tinea barbae in a tertiary Portuguese hospital: a 11-year audit. Mycoses.

[8] Mebazaa A, EL Oumari K, Ben Said M, Ghariani N, Denguezli M, Mili AF (2010). Tinea capitis in adults in Tunisia. Int J Dermatol.

[9] Lova-Navarro M, Gómez-Moyano E, Pilar LM, Fernandez-Ballesteros MD, Godoy-Díaz DJ, Vera-Casaño A (2016). Tinea capitis in adults in southern Spain A 17-year epidemiological study. Rev Iberoam Micol.

[10] Khosravi AR, Shokri H, Vahedi G (2016). Factors in etiology and predisposition of adult tinea capitis and review of published literature. Mycopathologia.

[11] Park SK, Park SW, Yun SK, Kim HU, Park J (2019). Tinea capitis in adults: a 18-year retrospective, single-centre study in Korea. Mycoses.

[12] Yu J, Chen W, Wan Z, Li R (2004). Adult tinea capitis due to *Trichophyton violaceum* in China. Mycopathologia.

[13] Liang G, Zheng X, Song G, Zhang M, Liu J, Zang X (2020). Adult tinea capitis in China: a retrospective analysis from 2000 to 2019. Mycoses.

[14] Lee JYY, Hsu ML (1991). Tinea capitis in adults in southern Taiwan. Int J Dermatol.

[15] Cervetti O, Albini P, Arese V, Ibba F, Novarino M, Panzone M (2014). Tinea capitis in adults. Adv Microbiol.

[16] Ooka S, Kashima M, Kubota Y, Noguchi A, Kawai S, Nakamura Y (2000). A case of black dot ringworm with a review of Japanese cases. J Dermatol.

[17] Tangjaturonrusamee C, Piraccini BM, Vincenzi C, Starace M, Tosti A (2011). Tinea capitis mimicking folliculitis decalvans. Mycoses.

[18] Tirado-Sánchez A, Estrada-Caraveo Y, Saldaña M, Bonifaz A (2019). Adult tinea capitis: a clinical entity in increasing frequency. Curr Fungal Infect Rep.

[19] Souissi A, Ben Lagha I, Toukabri N, Mama M, Mokni M (2018). Morse code-like hairs in tinea capitis disappear after successful treatment. Int J Dermatol.

[20] Elghblawi E (2016). Idiosyncratic findings in trichoscopy of tinea capitis: comma, zigzag hairs, corkscrew, and morse code-like Hair. Int J Trichol.

[21] Elewski BE (2000). Tinea capitis: a current perspective. J Am Acad Dermatol.

[22] Genedy RM, Sorour OA, Elokazy MAW (2020). Trichoscopic signs of tinea capitis: a guide for selection of appropriate antifungal. Int J Dermatol.

[23] Han D, Liu Y, Zhu J, Li L, Zhang Q (2016). Comparison of novel fluorescent staining and KOH microscopic examination in fungal measured by direct microscopy. Chin J Mycol.

[24] Gupta AK, Katz HI, Shear NH (1999). Drug interactions with itraconazole, fluconazole, and terbinafine and their management. J Am Acad Dermatol.

[25] Tey HL, Tan ASL, Chan YC (2011). Meta-analysis of randomized, controlled trials comparing griseofulvin and terbinafine in the treatment of tinea capitis. J Am Acad Dermatol.

[26] Group S (1999). Short duration treatment with terbinafine for tinea capitis caused by *Trichophyton* or *Microsporum* species. Br J Dermatol.

[27] Bouchara JP, Chaturvedi V. The curious case of “Case report” of infections caused by human and animal fungal pathogens: An educational tool, an online archive, or a format in need of retooling. Mycopathologia. (2018). 10.1007/s11046-018-0314-1 30570717

